# Supplementary material for: Subtle differences in the pathogenicity of SARS-CoV-2 variants of concern B.1.1.7 and B.1.351 in rhesus macaques
Source: bioRxiv. 2021 May 7:2021.05.07.443115. Preprint. [Version 1] doi: 10.1101/2021.05.07.443115 (PMC8357058; doi:10.1101/2021.05.07.443115)
Supplement: 1 [file NIHPP2021.05.07.443115V1-supplement-1.pdf]

bioRxiv preprint doi: https://doi.org/10.1101/2021.05.07.443115; this version posted May 7, 2021. The copyright holder for this preprint (which was not certified by peer review) is the author/funder. This article is a US Government work. It is not subject to copyright under 17 USC 105 and is also made available for use under a CC0 license.

Supplemental materials

Subtle differences in the pathogenicity of SARS-CoV-2 variants of concern B.1.1.7 and B.1.351 in rhesus macaques

Vincent J. Munster[1], Meaghan Flagg[1], Manmeet Singh[1], Brandi N. Williamson[1], Friederike Feldmann[2], Lizzette Pérez-Pérez[1], Beniah Brumbaugh[1], Myndi G. Holbrook[1], Danielle R. Adney[1], Atsushi Okumura[1], Patrick W. Hanley[2], Brian J. Smith[2], Jamie Lovaglio[2], Sarah L. Anzick[3], Craig Martens[3], Neeltje van Doremalen[1], Greg Saturday[2], and Emmie de Wit[1*]

[1]Laboratory of Virology, [2]Rocky Mountain Veterinary Branch and [3]Research Technologies Branch, Division of Intramural Research, National Institute of Allergy and Infectious Diseases, National Institutes of Health, Hamilton, MT, United States of America

bioRxiv preprint doi: https://doi.org/10.1101/2021.05.07.443115; this version posted May 7, 2021. The copyright holder for this preprint (which was not certified by peer review) is the author/funder. This article is a US Government work. It is not subject to copyright under 17 USC 105 and is also made available for use under a CC0 license.

Table S1. Genome changes observed in virus inoculum and 2dpi BAL samples as compared to reference sequence. Changes are indicated in allelic fraction calculated after filtering as indicated in Methods.

					Animal no.			
**ORF**	**a.a. change**	**inoculum**	**RM1**	**RM2**	**RM3***	**RM4**	**RM5**	**RM6**
**D614G**								
nsp6	H3631H	<0.1	<0.1	0.98	0	<0.1	<0.1	<0.1
**B.1.1.7**		**inoculum**	**RM7**	**RM8**	**RM9**	**RM10**	**RM11**	**RM12**
nsp6	D165G	0.14	<0.1	<0.1	0.16	<0.1	<0.1	<0.1
nsp6	F181V	<0.1	<0.1	<0.1	<0.1	<0.1	0.10	<0.1
nsp6	L257F	0.18	0.22	0.23	0.28	0.22	0.20	0.18
nsp7	V11I	0.13	0.22	0.21	<0.1	0.28	0.21	0.45
**B.1.351**		**inoculum**	**RM13**	**RM14****	**RM15**	**RM16**	**RM17**	**RM18**
nsp5	P252L	0.17	0.32	<0.1	0.31	0.28	0.34	0.30
nsp6	L257F	0.57	0.47	0.64	0.51	0.58	0.48	0.53

*Poor read coverage

**Marginal read coverage

bioRxiv preprint doi: https://doi.org/10.1101/2021.05.07.443115; this version posted May 7, 2021. The copyright holder for this preprint (which was not certified by peer review) is the author/funder. This article is a US Government work. It is not subject to copyright under 17 USC 105 and is also made available for use under a CC0 license.

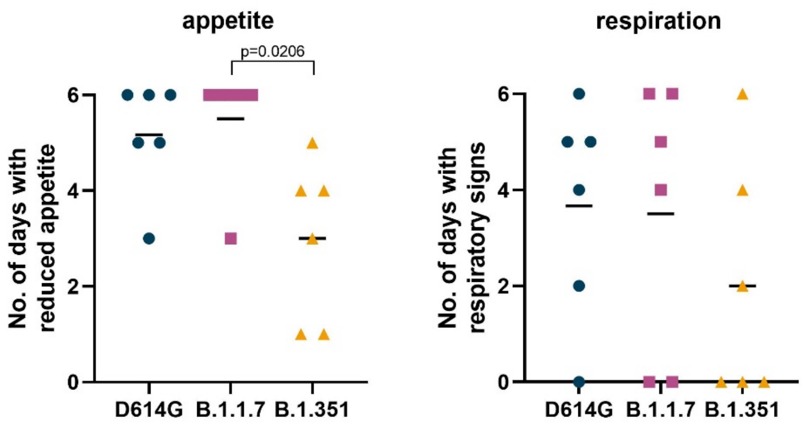

Figure S1. Clinical disease observed in rhesus macaques inoculated with D614G, B.1.1.7 and B.1.351. Three groups of six adult rhesus macaques were inoculated with SARS-CoV-2 variants D614G, B.1.1.7 or B.1.351. After inoculation, animals were observed for disease signs and scored according to a pre-established clinical scoring sheet. The number of days an animal showed reduced appetite (left panel) or changes in respiration pattern (right panel) are indicated. Statistical analysis was performed using a Kruskal-Wallis test with Dunn's multiple comparisons; p-values <0.05 are indicated.

bioRxiv preprint doi: https://doi.org/10.1101/2021.05.07.443115; this version posted May 7, 2021. The copyright holder for this preprint (which was not certified by peer review) is the author/funder. This article is a US Government work. It is not subject to copyright under 17 USC 105 and is also made available for use under a CC0 license.

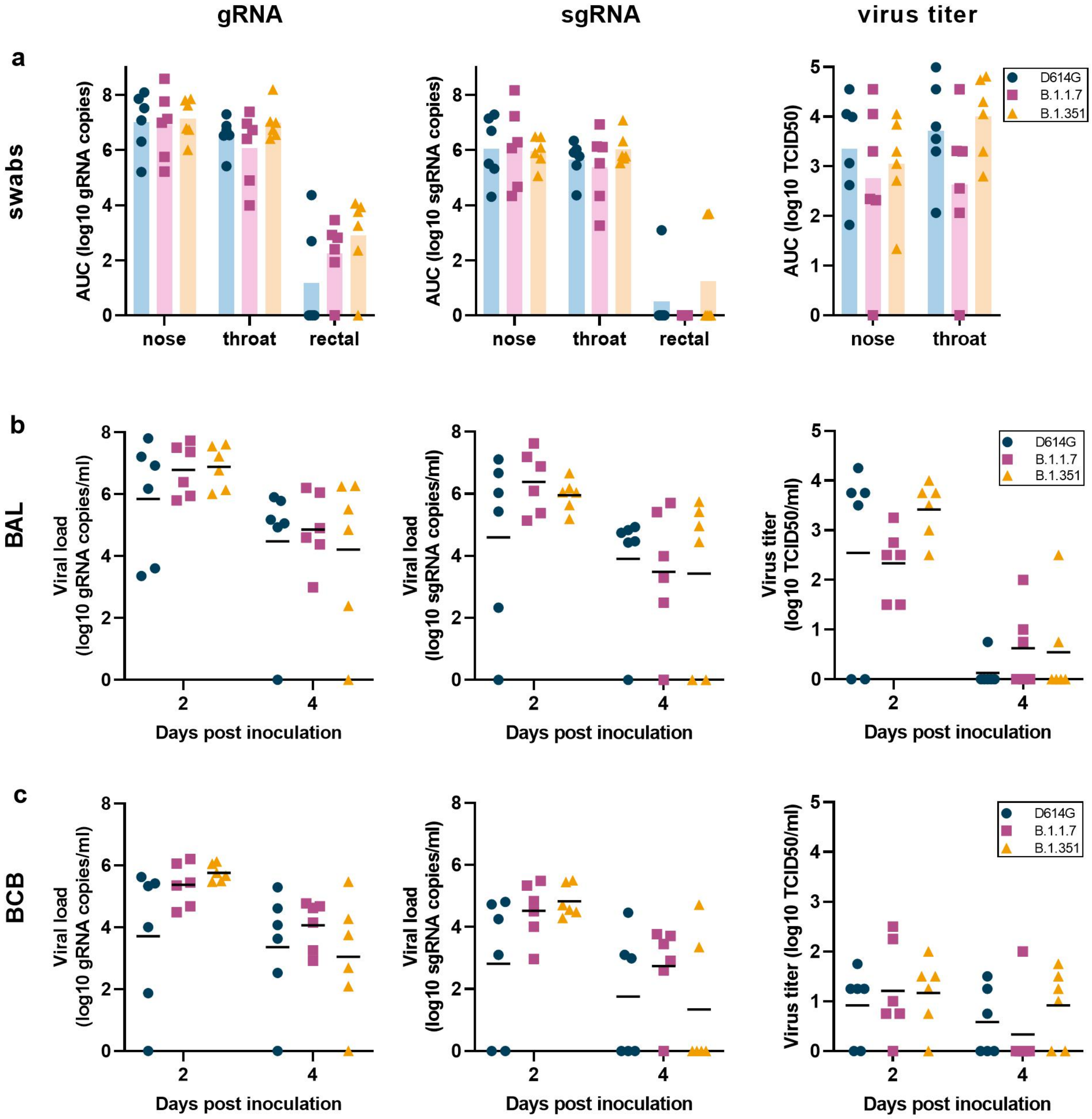

Figure S2. SARS-CoV-2 viral loads and virus titers in swabs, bronchoalveolar lavages and bronchial cytology brushes. Three groups of six adult rhesus macaques were inoculated with SARS-CoV-2 variants D614G, B.1.1.7 or B.1.351. After inoculation, clinical exams were performed on 2, 4, and 6 dpi during which nose, throat, and rectal swabs were collected. qRT-PCR was performed to detect genomic (left column) and subgenomic RNA (middle column), and virus titration was performed to detect levels of infectious virus (right column) and the Area Under the Curve (AUC) was calculated as an indication of the total amount of virus shed in these samples. Swabs (a), bronchoalveolar lavages (BAL) (b) and bronchial cytology brush (BCB) samples (c) were collected on 2 and 4 dpi and analyzed for the presence of gRNA, sgRNA and infectious virus. Lines indicate the mean. Bars (a) and lines (b, c) indicate the mean. Statistical analysis was performed using a Kruskal-Wallis test with Dunn's multiple comparisons tests (a) or a 2-way ANOVA with Tukey's multiple comparisons test (b, c); no p-values <0.05 were found.

bioRxiv preprint doi: https://doi.org/10.1101/2021.05.07.443115; this version posted May 7, 2021. The copyright holder for this preprint (which was not certified by peer review) is the author/funder. This article is a US Government work. It is not subject to copyright under 17 USC 105 and is also made available for use under a CC0 license.

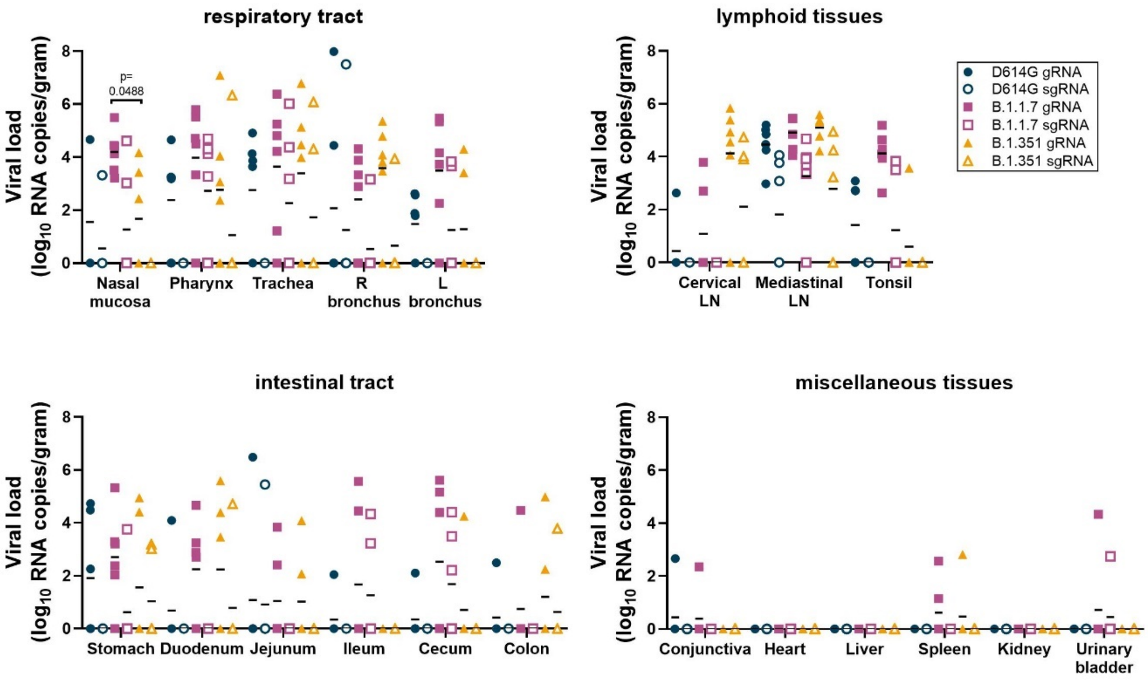

Figure S3. Viral loads in tissues collected from D614G, B.1.1.7 or B.1.351-inoculated rhesus macaques on 6 dpi. Three groups of six adult rhesus macaques were inoculated with SARS-CoV-2 variants D614G, B.1.1.7 or B.1.351. On 6 dpi, all animals were euthanized and necropsies were performed. Samples were collected from many different organs and analyzed for the presence of gRNA (closed symbols) and sgRNA (open symbols). Lines indicate the mean. Statistical analysis was performed using a 2-way ANOVA with Tukey's multiple comparisons test; p-values <0.05 are indicated.

bioRxiv preprint doi: https://doi.org/10.1101/2021.05.07.443115; this version posted May 7, 2021. The copyright holder for this preprint (which was not certified by peer review) is the author/funder. This article is a US Government work. It is not subject to copyright under 17 USC 105 and is also made available for use under a CC0 license.

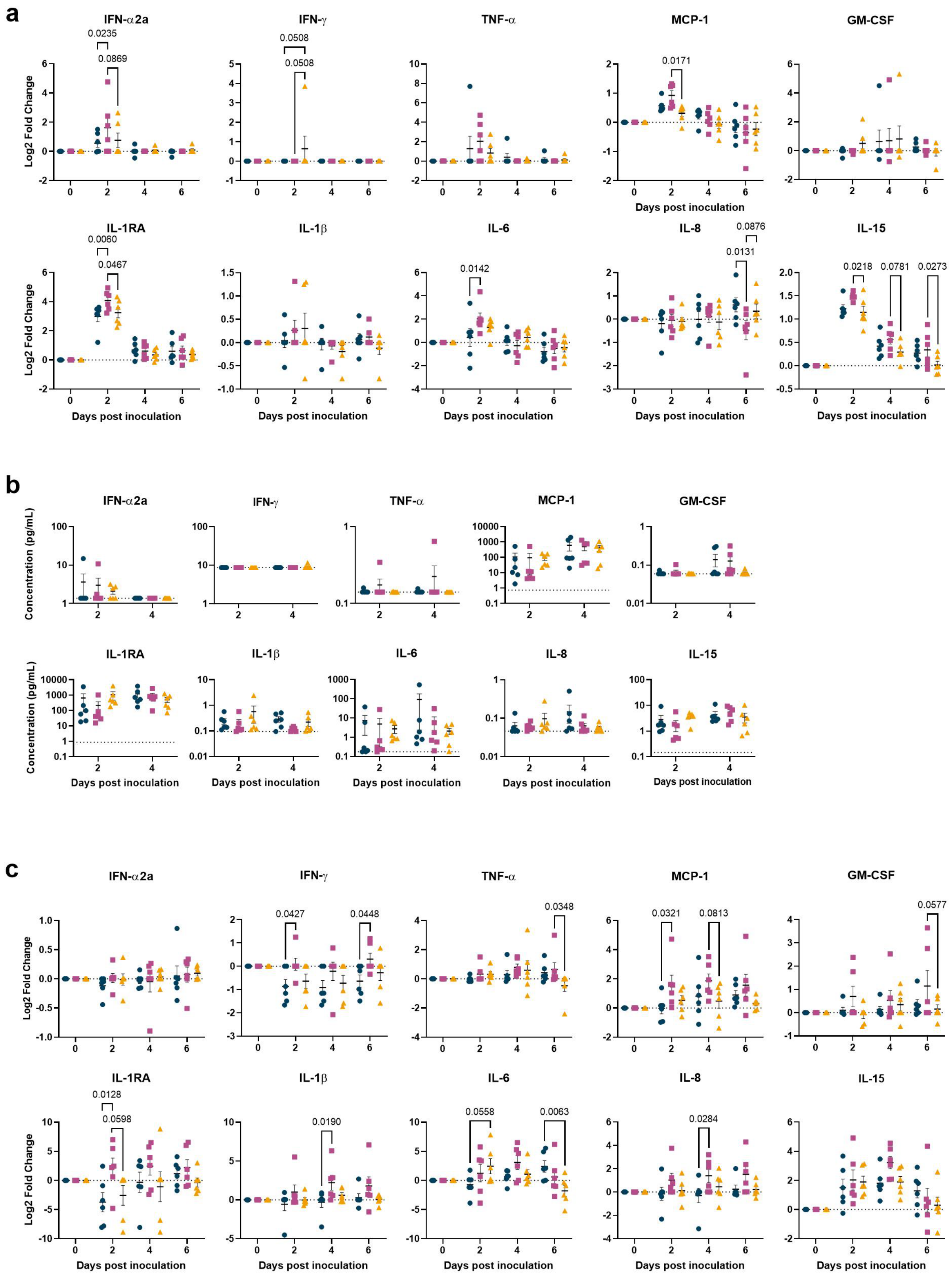

bioRxiv preprint doi: https://doi.org/10.1101/2021.05.07.443115; this version posted May 7, 2021. The copyright holder for this preprint (which was not certified by peer review) is the author/funder. This article is a US Government work. It is not subject to copyright under 17 USC 105 and is also made available for use under a CC0 license.

Figure S4. Cytokine and chemokine responses in rhesus macaques inoculated with a D614G, B.1.1.7 or B.1.351 isolate of SARS-CoV-2. The concentration of 10 different cytokines and chemokines were measured in serum (a), BAL (b), and nasal samples (c) at different timepoints before and after inoculation. Fold 2 log changes were calculated for samples where baseline (0 dpi) values were available (a, c); in the absence of a baseline sample concentrations are plotted in pg/mL (b). Blue circles: D614G; pink squares: B.1.1.7; yellow triangles: B.1.351. Dotted lines indicate no change from baseline (a, c) or the maximum lower limit of detection calculated across plates (b). Statistical analysis was performed using a 2-way ANOVA with Tukey's multiple comparisons test; p-values <0.05 are indicated.
